# Active self-touch restores bodily proprioceptive spatial awareness following disruption by ‘rubber hand illusion'

**DOI:** 10.1098/rspb.2023.1753

**Published:** 2024-01-17

**Authors:** Antonio Cataldo, Damiano Crivelli, Gabriella Bottini, Hiroaki Gomi, Patrick Haggard

**Affiliations:** ^1^ Institute of Cognitive Neuroscience, University College London, Alexandra House, 17 Queen Square, London WC1N 3AZ, UK; ^2^ Department of Brain and Behavioural Sciences, University of Pavia, Pavia, Italy; ^3^ NeuroMi, Milan Centre for Neuroscience, Milan, Italy; ^4^ Cognitive Neuropsychology Centre, ASST Grande Ospedale Metropolitano Niguarda, Milano, Italy; ^5^ NTT Communication Science Laboratories, Nippon Telegraph and Telephone Corporation, Atsugi, Japan

**Keywords:** self-touch, bodily self-awareness, body ownership, rubber hand illusion, voluntary action

## Abstract

Bodily self-awareness relies on a constant integration of visual, tactile, proprioceptive, and motor signals. In the ‘rubber hand illusion' (RHI), conflicting visuo-tactile stimuli lead to changes in self-awareness. It remains unclear whether other, somatic signals could compensate for the alterations in self-awareness caused by visual information about the body. Here, we used the RHI in combination with robot-mediated self-touch to systematically investigate the role of tactile, proprioceptive and motor signals in maintaining and restoring bodily self-awareness. Participants moved the handle of a leader robot with their right hand and simultaneously received corresponding tactile feedback on their left hand from a follower robot. This self-touch stimulation was performed either before or after the induction of a classical RHI. Across three experiments, active self-touch delivered after—but not before—the RHI, significantly reduced the proprioceptive drift caused by RHI, supporting a restorative role of active self-touch on bodily self-awareness. The effect was not present during involuntary self-touch. Unimodal control conditions confirmed that both tactile and motor components of self-touch were necessary to restore bodily self-awareness. We hypothesize that active self-touch transiently boosts the precision of proprioceptive representation of the touched body part, thus counteracting the visual capture effects that underlie the RHI.

## 1. Introduction

James' description of the ‘same old body, always there' [1], highlights that our own body is the most familiar object in our mental life. However, it remains unclear how individual sensory experiences produce a general bodily self-awareness. In the ‘rubber hand illusion' (RHI) [2], the participant receives tactile stimulation on her unseen hand, while seeing the same stimulation performed on a fake, rubber hand. When visual and tactile stimulations are synchronous, participants often report feeling that the rubber hand is theirs, and part of their own body: the fake hand is ‘embodied'. Asynchronous stimulation is commonly used as a control condition. The RHI has been assessed qualitatively [3], and also using quantitative proxy measures in which participants report the position of their unseen hand [4]. Participants tend to perceive their hand as shifted towards the location where they saw the fake hand. Crucially, this tendency is stronger in the synchronous than the asynchronous condition. Proprioceptive drift is therefore a quantitative measure of how the visual experience in the RHI influences spatial perception of the body [5], and may also have value as an implicit measure of the wider changes in bodily self-awareness that occur during the RHI.

The RHI arises because visual and proprioceptive information about the hand conflict. One aspect of the brain's resolution of this conflict is the generation of a synthetic estimate of the position of the hand, made by combining visual and proprioceptive signals after weighting according to their respective reliabilities [6]. Because vision is often more reliable than proprioception, this estimate is typically biased towards the location where the rubber hand was viewed. In these cases, vision dominates proprioception, but when proprioception is more reliable than vision, this relation is reversed [7]. For example, bodily self-awareness remains in the dark, when vision is absent. The non-visual aspects of body self-awareness are strongly present in the act of self-touch. Self-touch is common in humans and many other animals. For example, self-touch occurs during purposive actions such as grooming, pleasurable self-stimulation and thermoregulation behaviours, and also occurs incidentally during behaviours such as feeding and bimanual object handling. A long phenomenological interest in self-touch [8,9] focuses on the deep integration of motor and tactile signals [10–12], and suggests that the resulting sensorimotor contingencies may underpin the development of a coherent, stable sense of a bodily self.

Some studies have combined self-touch and RHI approaches. First, Ehrsson *et al*. [13] used a modified form of self-touch to develop a proprioceptive analogue of the visual RHI, sometimes known as somatic RHI. An experimenter tapped the participant's finger passively against a rubber hand, while they received synchronous taps on their own hand. This produced a convincing illusion of tapping one's own hand, and a proprioceptive drift in the perceived position of one's own tapped hand towards the rubber hand. This result suggests that tactile, motor and proprioceptive signals associated with self-touch make an important contribution to body awareness. This initial result was subsequently confirmed and extended [14–18]. Importantly, these studies have no visual component—participants are typically blindfolded. It therefore remains unclear how the external visual perspective on one's own body (as in the classical RHI), and the intrinsic motor-somatosensory contribution (as in self-touch) might be combined.

We addressed this question by investigating whether self-touch can restore a disturbance in bodily awareness caused by visual RHI. This hypothesis is motivated by findings from clinical neuropsychology. Disturbance of body awareness [19] may occur in a number of neurological and psychiatric conditions. In a single-case study of somatoparaphrenia—a rare clinical condition in which body parts can be misattributed to others [20]—Van Stralen *et al*. [21] showed that actively self-touching the affected limb led to a remission of misattribution. Further, patients with hemianaesthesia showed improvements in tactile detection, localization and perceived intensity when the stimuli were delivered through self-touch compared with other forms of tactile stimulation [22,23]. Moreover, a recent study by Roel Lesur *et al.* [17] showed that active self-touch produces a stronger sense of body ownership compared with touch by another person, even when significant temporal mismatches were introduced in a visuo-tactile stimulation. This finding led the authors to suggest that self-touch might play a crucial role in sustaining normal bodily awareness in contexts that might otherwise tend to produce feelings of disembodiment. Thus, in addition to testing the restorative role of self-touch on bodily awareness, we also investigated whether a brief self-touch stimulation taking place *before* the RHI could have a protective effect on participants' bodily awareness.

We used two robotic arms in a leader-follower configuration to create an artificial self-touch condition [24,25] suitable for investigating the influence of self-touch on body awareness. By moving the handle of the leader robot with their right hand, participants were able to simultaneously feel a corresponding tactile feedback on their left forearm from the follower robot. Thanks to this mediated self-touch set-up, and in contrast to the direct skin–skin contact that occurs during everyday self-touch, the right-hand movement did not provide any direct information about the left hand's spatial location. We could therefore use the perceived position of the left hand as a measure of bodily self-awareness. We also collected self-report measures in each experiment.

We therefore report a systematic series of three experiments based on power calculation, replication and preregistration (Methods), to investigate the role of self-touch in bodily awareness. In Experiment 1, we explored the hypothesis that self-touch has a restorative effect on the altered body awareness caused by immediately preceding RHI. In Experiment 2, we instead investigated if self-touch has a protective effect on body self-awareness by asking the participant to perform a self-touch stimulation immediately *before* inducing the RHI. Importantly, in both experiments we also implemented passive self-touch conditions, in which the right hand of the participant was passively moved by the experimenter while stroking the left forearm [24,25]. By comparing effects of active and passive self-touch, we could therefore investigate whether motor signals play a distinctive role in body awareness. Finally, Experiment 3 used a larger sample estimated *a priori* by power analysis of results of Experiment 1 and included additional unimanual controls to investigate the separate contributions of movement and touch alone to the combined effect of self-touch on body awareness.

## 2. Results

### (a) Does self-touch have a restorative effect on bodily self-awareness?

**Figure 1.**
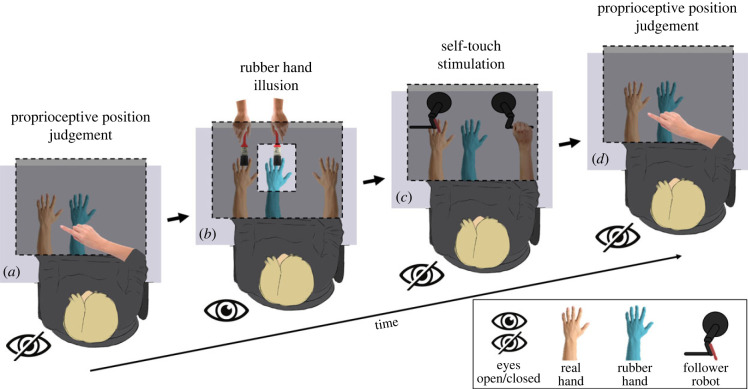
Experimental set-up. Participants sat at a desk, resting both their arms on the surface. A rubber hand was placed to the right of real left hand. The participants' left arm and the robotic set-up were covered by a foamboard screen and remained unseen throughout the entire experiment. The silicone glove was instead clearly visible through an aperture in the foamboard. (*a*,*d*) Baseline proprioceptive judgement. At the beginning and the end of each trial, participants were asked to close the eyes and to use their right index finger to produce a ballistic movement to point to the location immediately above the centre of their left wrist. Pointing performance was measured using a webcam suspended above the workspace. (*b*) RHI induction. The experimenter sat opposite the participant and used the two identical brushes to stroke homologous points of the participants' left hand and the cosmetic glove either synchronously or asynchronously for one minute, while the participant kept their gaze on the rubber hand. (*c*) Self-touch stimulation. Self-touch was performed immediately after (Experiments 1 and 3) or before (Experiment 2) the RHI stimulation using two six-degrees-of-freedom robotic arms coupled in a leader-follower system. The participants were asked to close their eyes and move the leader robot with their right hand. The participants' movement was reproduced by the follower robot thus generating corresponding gentle strokes from on the back of the participants' left middle.

**Figure 2.**
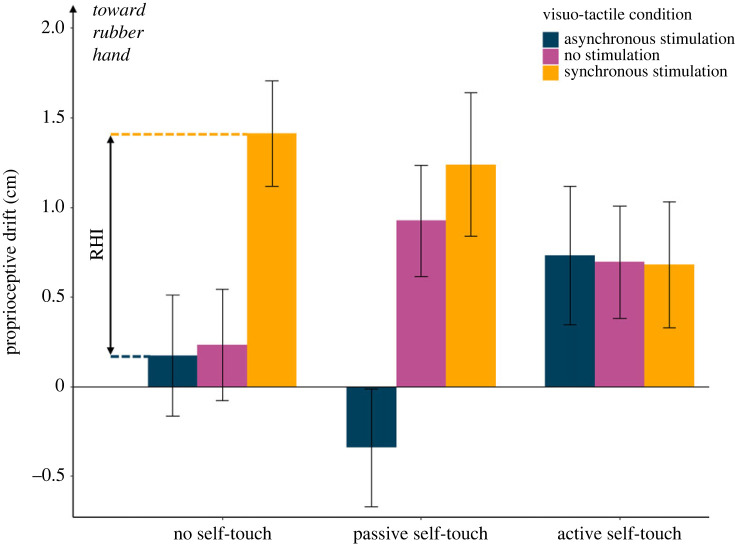
Results from Experiment 1. Both in the no self-touch and in the passive self-touch conditions, participants showed significantly larger proprioceptive drift in the synchronous compared with the asynchronous RHI condition. By contrast, following active self-touch condition, the three visuo-tactile conditions were virtually identical, suggesting a restorative effect of self-touch on bodily awareness. The error bars represent the s.e.m.

In Experiment 1, we tested whether brief self-touch stimulation immediately *after* the induction of the RHI could mitigate the effects of a previous RHI on body awareness. Participants (*n* = 16, based on an *a priori* power analysis, see Methods and electronic supplementary material) made a baseline proprioceptive judgement at the beginning of each trial by closing their eyes and pointing with their right hand to the location of their left hand (figure 1*a* and Methods). Next, they received one of three visuo-tactile stimulation conditions: synchronous, asynchronous or no stimulation (figure 1*b* and Methods). The RHI induction phase lasted for 60 s and was followed by one of three self-touch stimulations: active self-touch, passive self-touch and no self-touch. The self-touch stimulation was performed using two coupled robots that implemented a mediated form of self-touch and lasted for 15 s (see [24,25] and figure 1*c*). Immediately after the self-touch stimulation, the participants made a second proprioceptive judgement. Proprioceptive drift [2] was computed as the difference between the final and the baseline proprioceptive judgement on each trial. Figure 2 shows the individual and average proprioceptive drift results in each condition of Experiment 1.

If self-touch restored body awareness, then the proprioceptive drift caused by RHI should be reduced if RHI induction is immediately followed by self-touch. In line with this hypothesis, a 3 (visuo-tactile stimulation: synchronous, asynchronous, no stimulation) × 3 (self-touch condition: active, passive, no self-touch) repeated measures ANOVA on the data from Experiment 1 showed a significant main effect of visuo-tactile stimulation induction (*F*_2,__30_ = 11.49, *p* < 0.001, $\eta_{p}^{2}=0.0434$), no main effect of self-touch condition (*F*_2,__30_ = 0.09, *p* = 0.914, $\eta_{p}^{2}=0.006$), but a marginally significant interaction between factors (*F*_4,__60_ = 2.49, *p* = 0.052, $\eta_{p}^{2}=0.143$). The trend was explained by the difference between the synchronous and the asynchronous in the passive (synchronous: mean = 1.24, s.d. = 1.60; asynchronous: mean = − 0.34, s.d. = 1.32; *t*_15_ = 3.48; *p* = 0.003; Cohen's *d* = 0.870) and no self-touch (synchronous: mean = 1.41, s.d. = 1.18; asynchronous: mean = 0.18, s.d. = 1.36; *t*_15_ = 3.09; *p* = 0.007; Cohen's *d* = 0.772) conditions. By contrast, the active self-touch condition was virtually identical across all the three visuo-tactile stimulation conditions (synchronous: mean = 0.68, s.d. = 1.40; asynchronous: mean = 0.73, s.d. = 1.55; no stimulation: mean = 0.69, s.d. = 1.25; *p* ≥ 0.929 in all cases).

Previous studies suggest that proprioceptive drift and subjective reports of RHI reflect different constructs of bodily awareness [4,26–29]. Thus, although proprioceptive drift constituted the main dependent variable in this study, self-reported measures of the participants' subjective experience of the RHI were also collected in each experiment. In particular, at the end of every trial of each condition, participants indicated their level of agreement with a series of statements from an established bodily awareness questionnaire (adapted from [30]) (electronic supplementary material).

The self-report data showed a significant main effect of visuo-tactile stimulation for each questionnaire item (*p* < 0.003 in all cases) (electronic supplementary material, figure S1 and electronic supplementary material, tables S1 and S2). Neither the main effect of self-touch condition, nor the interaction between self-touch condition and visual-tactile stimulation were significant in any of the questionnaire items (*p* > 0.255 in all cases).

Thus, results from Experiment 1 provided some trend evidence that active self-touch immediately after a RHI stimulation might mitigate the altered bodily awareness induced by the RHI, supporting a restorative role of active self-touch on the proprioceptive component of bodily self-awareness.

### (b) Does self-touch have a protective effect on bodily awareness?

**Figure 3.**
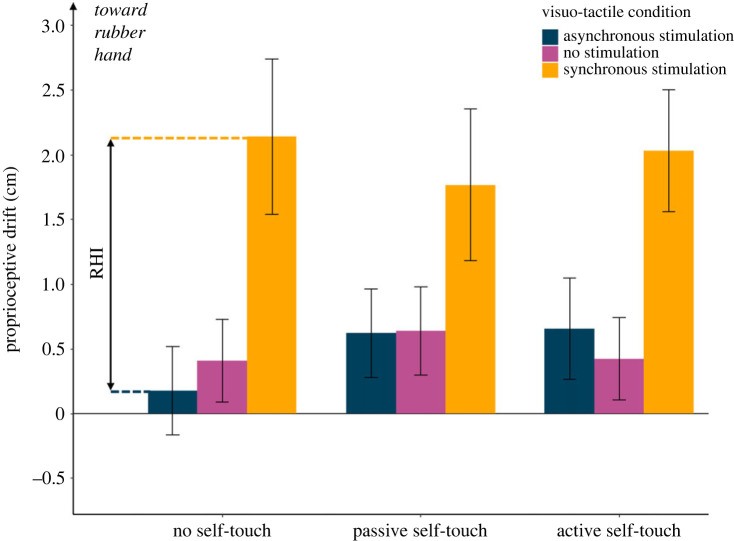
Results from Experiment 2. Self-touch delivered before the induction of the RHI had no effect on proprioceptive drift measures of RHI, ruling out the hypothesis that self-touch has a protective effect on bodily awareness. The error bars represent the s.e.m.

In Experiment 2, we tested in a new set of participants (*n* = 16) whether a brief self-touch stimulation performed *before* induction of the RHI had a protective effect on bodily self-awareness, by anticipatorily reducing susceptibility to the RHI. The experimental design was identical to Experiment 1, except that the order of self-touch stimulation and RHI induction was inverted. A protective effect on bodily self-awareness would be evidenced by reduction of the participants' proprioceptive drift after active, or even passive, self-touch, but not after the control no self-touch condition. Contrary to this hypothesis, a 3 (visuo-tactile stimulation: synchronous, asynchronous, no stimulation) × 3 (self-touch condition: active, passive, no self-touch) repeated measures ANOVA showed a significant main effect of visuo-tactile stimulation (*F*_1.37,20.57_ = 9.09, *p* = 0.004, $\eta_{p}^{2}=0.377$), but no main effect of self-touch condition (*F*_2,30_ = 0.69, *p* = 0.510, $\eta_{p}^{2}=0.044$) nor interaction between factors (*F*_4,60_ = 1.14, *p* = 0.347, $\eta_{p}^{2}=0.071$) (figure 3). The main effect of visuo-tactile stimulation was explained by a significantly higher proprioceptive drift in the synchronous RHI condition (mean = 2.06, s.d. = 2.14) compared with both the asynchronous (mean = 0.59, s.d. = 1.38; *t*_15_ = 3.32, *p* = 0.005; Cohen's *d* = 0.830) and the no stimulation condition (mean = 0.56, s.d. = 1.31; *t*_15_ = 3.10, *p* = 0.007; Cohen's *d* = 0.775), as expected.

Again, the self-report data showed a significant main effect of visuo-tactile stimulation for each questionnaire item (*p* < 0.018 in all cases), but no main effect of self-touch condition, nor interaction between factors (*p* > 0.050 in all cases) (electronic supplementary material, figure S2 and tables S3 and S4).

Thus, we found no evidence that performing a brief self-touch stimulation has any *protective* effect against changes in body awareness induced by a subsequent RHI.

### (c) Do unimodal components of self-touch independently affect bodily self-awareness?

Experiment 3 aimed to replicate and further investigate the results of Experiment 1. The sample size was estimated by an *a priori* power calculation based on effects in Experiment 1 (electronic supplementary material, Methods), and the experiment was preregistered (https://osf.io/ygqnf). Further conditions were included to investigate *why* active self-touch might restore altered bodily awareness: was restoration driven by active movement of the right hand, by tactile stimulation of the left hand, or did it require the combination of both factors? As in Experiment 1, RHI induction was always followed by a self-touch intervention. However, self-touch conditions were provided by a factorial combination of right-hand movement (present, absent) and left-arm touch (present, absent). The conditions where movement and touch were both present or both absent were identical to the active self-touch and no self-touch conditions of Experiment 1, respectively. The other two factorial combinations (movement only and touch only) served as control conditions to investigate whether either the motor or the tactile component of self-touch alone was sufficient to influence bodily awareness.

**Figure 4.**
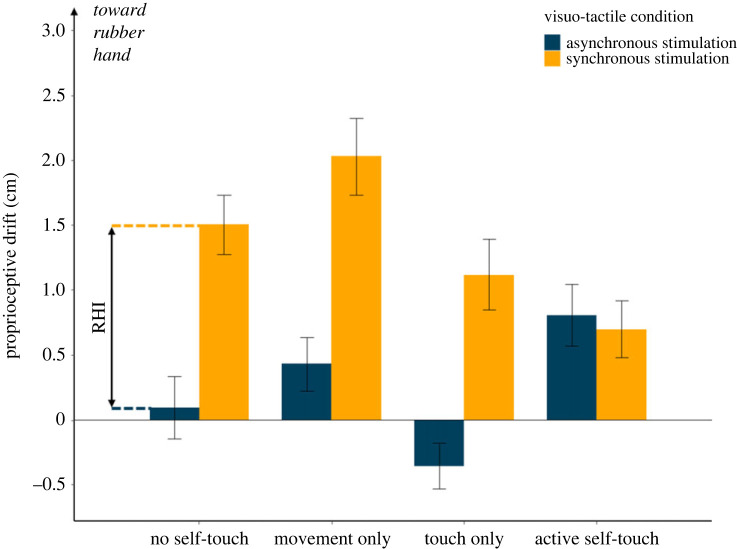
Results from Experiment 3. Motor and tactile components of self-touch were organized according to a 2 × 2 factorial arrangement (movement absent/present; touch absent/present), and followed immediately after synchronous or asynchronous visuo-tactile stimulation. Unimodal motor and tactile stimulation conditions and no self-touch condition all showed a significant RHI, defined as the difference in proprioceptive drive between the synchronous and asynchronous visuo-tactile conditions. After active self-touch involving both movement and touch components the two visuo-tactile conditions were not statistically different. This replicates the results from Experiment 1 and confirms the role of active self-touch in restoring bodily awareness. Error bars represent s.e.m.

We predicted (https://osf.io/ygqnf) a significant interaction between the visuo-tactile stimulation factor (synchronous, asynchronous), the movement factor (present, absent) and the touch factor (present, absent). This prediction was based on the hypothesis that bodily awareness, as indicated by a reduction in proprioceptive drift associated with RHI, would be restored only by active self-touch. In particular, we hypothesized that the magnitude of the RHI-induced proprioceptive drift would be smaller when followed by an active self-touch condition involving both movement and touch, relative to other conditions. A 2 (visuo-tactile stimulation: synchronous, asynchronous) × 2 (movement: present, absent) × 2 (touch: present, absent) repeated measures ANOVA showed main effects of visuo-tactile stimulation (*F*_1,__27_ = 50.75, *p* < 0.001, $\eta_{p}^{2}=0.653$), touch (*F*_1,27_ = 12.18, *p* = 0.002, $\eta_{p}^{2}=0.311$) and movement (*F*_1,27_ = 4.05, *p* = 0.054, $\eta_{p}^{2}=0.130$), and the predicted significant three-way interaction between all factors (*F*_1,27_ = 18.51, *p* < 0.001, $\eta_{p}^{2}=0.407$). There was no mean difference in proprioceptive drift between synchronous and asynchronous RHI following active self-touch (synchronous: mean = 0.70, s.d. = 1.16; asynchronous: mean = 0.81, s.d. = 1.26; *t*_27_ = 0.46; *p* = 0.647; Cohen's *d* = 0.088). By contrast, the difference between proprioceptive drift in synchronous and asynchronous RHI conditions was significant in all the other conditions (movement only: synchronous: mean = 2.02, s.d. = 1.56; asynchronous: mean = 0.43, s.d. = 1.09; *t*_27_ = 5.21, *p* < 0.001; Cohen's *d* = .985; touch only: synchronous: mean = 1.11, s.d. = 1.45; asynchronous: mean = − 0.35, s.d. = 0.93; *t*_27_ = 5.00, *p* < 0.001; Cohen's *d* = .944, no movement no touch: synchronous: mean = 1.50, s.d. = 1.21; asynchronous: mean = 0.099, s.d. = 1.276; *t*_27_ = 5.59, *p* < 0.001; Cohen's *d* = 1.057) (figure 4). Thus, only the combined motor and tactile signals that characterize normal self-touch were able to restore the changes in bodily self-awareness induced by RHI.

Finally, self-report data showed the main effect of visuo-tactile stimulation was significant for every questionnaire item (*p* < 0.011 in all cases), except control question number five (*It felt as if my (real) left hand were turning ‘rubbery'*, *p* = 0.101). Similar to the results of the previous experiments, the main effect of self-touch condition was non-significant (*p* > 0.111 in all cases). However, there was a significant interaction for question two (*It felt as if I was looking directly at my left hand*; *p* < 0.001), four (*The rubber hand began to resemble my (real) left hand*; *p* = 0.006), and six (*It seemed as though the touch I felt was caused by the paintbrush touching the rubber hand*; *p* = 0.006) (electronic supplementary material, figure S3 and tables S5 and S6). All the interactions were explained by the fact that, similar to the proprioceptive drift results, the difference between synchronous and asynchronous visuo-tactile stimulations was significantly larger in the no self-touch compared with the active self-touch condition (electronic supplementary material).

Thus, ‘complete’ active self-touch could restore altered body awareness induced by the RHI, but the individual motor and tactile components of self-touch could not when these were presented alone. This result suggests that correlation between movement and sensory stimulation associated with active self-touch is crucial for restorative effects on body awareness. Only the unique sensorimotor integration of motor and tactile information during active self-touch can restore the altered bodily self-awareness induced by the RHI.

## 3. Discussion

Across three experiments, we investigated the relationship between bodily self-awareness and self-touch through the RHI. Based on previous studies [15,17,21–23], we hypothesized that active self-touch could contribute to body awareness. We tested this by first inducing a well-established multi-sensory alteration of body awareness, namely the RHI, and then assessing whether self-touch could either restore body awareness after these alterations or protect against subsequent alterations. Importantly, we measured body awareness via a simple, quantitative proxy measure with a clear sensory-physiological interpretation, namely the perceived position of the stimulated hand, as well as with self-reports.

As we predicted, self-touch indeed influenced body awareness. Experiments 1 and 3 showed that active self-touch *after* the induction of a classic RHI reduced the proprioceptive drift caused by the RHI. This complements previous findings [13,15] showing that a ‘somatic RHI' could influence body self-awareness. Crucially, our results show that active self-touch is also able to *restore* bodily self-awareness after previous alterations induced by visual RHI.

To our knowledge, this is the first study showing restorative effects of self-touch on body representation and bodily self-awareness in healthy participants. However, some previous studies with neurological patients are consistent with our findings. For example, a single case study of a stroke patient with somatoparaphrenia [21] showed that self-touch on the affected limb induced a remission of limb ‘disownership'. Several previous studies investigated effects of self-touch in hemi-anaesthesia [22,23,31]. These studies suggest that the effects of self-touch operate through cross-modal attention [32]. Patients perceived tactile stimuli more reliably when these were self-delivered, compared with when they were delivered by another person. This ‘self-touch enhancement' [22] was explained by the movements of the unaffected hand increasing the salience of events on the affected hand, like an ‘attentional wand' [31]. Equally, explicitly drawing attention to the location of the real hand or the rubber hand can modulate the strength of RHI in healthy participants [33].

In our study, the salience of afferent signals from the left hand could equally be decreased by the visual-proprioceptive conflict of the RHI. Indeed, Bayesian theories of multi-sensory integration explain proprioceptive drifts induced by the RHI as a change in the relative weightings of visual information and of proprioceptive information about left-hand position. Loss of tactile sensitivity during RHI provides independent evidence that visual–tactile conflict can produce downweighting of somatosensory signals from the hand [34,35]. We suggest that subsequent active self-touch might restore a normal salience to the somatosensory signals from the stimulated left hand. In our case, we have measured the somatosensory signals that underlie proprioception rather than touch.

Normal skin-to-skin self-touch makes it difficult or impossible to separate the spatial locations of movement and of touch. As a result, the effects of motor-tactile association, and the effect of spatial attention both facilitate processing at the location of the touch, making effects of motor-tactile association and spatial attention difficult to dissociate. In contrast, our robot-mediated self-touch design distinguishes these two components for the first time. Our participants never saw the location of either hand and never directly touched one hand with the other. Participants had proprioceptive information from each limb to indicate the location of each hand, and they also experienced the strong association between the movements of the right hand and the touch on the left, in the active self-touch condition. Thus, in our set-up, the right hand's movement could potentially enhance the processing of proprioceptive signals arising from the left hand, without redirecting participants' attention to its location in space.

We also showed that the ‘restorative' effect of self-touch on body awareness was not due to movement alone, nor to touch alone, but to the unique combination of these individual signals that arises in self-touch. Ruling out ‘unimodal' explanations of self-touch effects is important for methodological reasons. For example, we measured body awareness by asking participants to point with their right hand to the location of their left hand. The simple act of moving the right arm during self-touch could potentially influence the control of these pointing movements [36] confounding motor repetition effects with our readout of body awareness. Similarly, receiving a tactile stimulus on a body part could have automatically redirected attention toward that spatial location, which could have also influenced judgements of hand position. However, these confounds are addressed by the unimodal motor and tactile conditions of Experiment 3 respectively. We found that only the multi-modal condition of active self-touch, but not the motor or tactile unimodal conditions, affected our measure of perceived left-hand location, thus controlling for these other components of sensory and motor processing, and ruling out these artefactual explanations.

Our design also distinguishes effects of self-touch from simple spatial averaging or spatial integration of the various events occurring during the experiment. Our main measure involved judgements of the proprioceptively perceived position of the left hand. Both the rubber hand, and the participant's right hand that administered active self-touch lay to the *right* of the participant's left hand (figure 1). The RHI therefore involves a rightward shift in the perceived position of the left hand, as the more reliable visual evidence for hand location dominates or captures less reliable proprioceptive information. Further, any general tendency for perceived hand position to drift spontaneously towards the midline over time irrespective of stimulation [37] is controlled for by the Asynchronous stimulation condition of the RHI. For example, active movements of the right hand might simply shift attention rightwards. Equally, the correlation between right-hand movement and left touch might produce a form of spatial attraction or spatial binding, where both events are assumed to be collocated, as is the case with everyday, unmediated self-touch. However, such effects would all produce *rightward* shifts of the perceived position of the left hand. In fact, our results clearly show that active self-touch reduces or even reverses the rightward shift in perceived position of the left hand. Our effects of self-touch thus operate in the opposite spatial direction to both the RHI and any general spatial attraction or spatial averaging effects between the hands. Thus, our results can only be explained by self-touch causing a strengthening of body awareness with respect to the left hand, pulling the perceived position back leftwards towards the actual location of the participants' left hand, rather than a spatial integration or averaging effect.

The perception of body part spatial location is a very important part of bodily self-awareness [38]. However, there may be additional components that also contribute to the sense of one's own body [3]. In our study, participants also answered a series of explicit questions about body awareness. Our preregistered analyses mainly focused on proprioception because it has several desirable psychometric properties, including clear physical correlates, quantitative measurement, and a straightforward question. In this sense, experimental results using proprioceptive drift measures can be easier to interpret than results using multi-item questionnaires. The relation between proprioceptive drift and questionnaires remains unclear. This may partly reflect the heterogeneity of the questions used, and their psychometric response properties. Our analyses of the questionnaire data showed no effects of active self-touch on these items. However, dissociations between questionnaire responses and quantitative measures based on perceived position are not uncommon in the body awareness literature [4,26–29]. Recent accounts of somatoparaphrenia [39] consider the erroneous spatial representation of the limb position, due to a poor proprioceptive update, as the key component of the deficit. Measures of perceived location have the advantage of being implicit, and do not rely on participants' comprehension of the words used in body awareness questionnaires [3,40]. Further, theoretical considerations suggest that spatial localization is central to constructing a mental representation of one's own body [41].

Our measures of body awareness were based on proprioceptively perceived position of the left hand. Since the left hand did not move at any point during the experiment, the effects of self-touch on body awareness cannot reflect changes in proprioceptive stimulation or proprioceptive afferent signalling. Self-touch must therefore have changed a *central* state estimate of hand position based on an unchanging proprioceptive input from the left arm. Bayesian interpretations of the RHI [42–44] suggest that proprioceptive drift in the RHI could be reduced or reversed either by increasing the precision of proprioceptive signals of hand position, or by decreasing the precision of the visual signal [7]. For example, Chancel & Ehrsson [45] recently found that decreasing proprioceptive precision by tendon vibration leads to increased visual capture in RHI. The same mechanism could potentially explain the effects we have observed here. Self-touch could transiently boost precision of proprioceptive representation of the touched body part, thus reducing the visual capture effect of the RHI.

The precise physiological mechanisms by which self-touch could boost proprioceptive precision remains unclear. Close integration of tactile and proprioceptive information is found in many post-primary somatosensory cortical regions, such as posterior parietal cortex [46]. However, the absence of any changing proprioceptive input from the left hand during our experiment, together with the crucial additional role of voluntary movement of the right hand, both point to a neurophysiological mechanism beyond mere tactile-proprioceptive integration [47]. We suggest two additional mechanisms that could be in play. First, voluntary self-touch could involve a shift of central attention onto the touched hand, boosting proprioceptive precision. Second, our self-touch set-up could effectively function as a virtual tool. The movements of the right hand were transferred to tactile stimulations of the left hand as if the participant had touched themselves with a stick. Humans and monkeys can learn a model of the kinematic chain linking their hand position to the endpoint of a tool in such cases [48,49]. Once such a model is learned, our participants could potentially use the position of the right hand as an additional source of information about the position of the left hand, thus increasing proprioceptive precision. An explanation using model-based perceptual learning, in which the position of the right hand becomes a useful source of information about the position of the left hand, could therefore potentially contribute to our finding that self-touch restores body awareness after RHI (Experiments 1 and 3). However, it remains striking that this additional proprioceptive information does not protect against the distorting effects on proprioception of subsequent RHI (Experiment 2). Our conditions could be viewed as requiring participants to learn two successive and conflicting kinematic models regarding the position of the left hand: a visual one in the RHI phase and a proprioceptive one in the self-touch phase. Since the two models interfere, body awareness might reflect only the most recently learned model.

More generally, the role of voluntary self-touch in proprioceptive awareness [50,51] suggests therapeutic promise in restoring disturbances in body awareness that occur in a range of neurological and psychiatric diseases.

## 4. Limitations

One limitation of the present study is that we did not provide the skin-to-skin self-touch of everyday experience, but instead used a robot-mediated analogue of self-touch [24,25]. However, mediated self-touch is common during everyday life (e.g. hairbrushes), and maintains the natural statistics of sensory-motor associations [52]. Moreover, this modification was necessary to ensure that the position of the left hand (the key readout for our proprioceptive drift measures of bodily awareness) could not be trivially known from the combination of proprioceptive signals arising from the right arm, and the fact of skin-to-skin self-touch.

A further limitation concerns our suggestion that self-touch reduces the RHI by boosting precision of proprioceptive representation of the hand. We have suggested this mechanism based on theoretical grounds. It is also consistent with previous evidence from the neuropsychological literature [31]. However, the number of trials in each condition of our experimental design is too low to obtain a reliable estimate of proprioceptive precision. Therefore, the present data do not allow a strong, direct test of this hypothesis. Future experiments could collect more trials in a reduced number of conditions in order to make more reliable estimates of proprioceptive precision, and thus test the hypothesis directly.

## 5. Material and methods

### (a) Participants

A total of 60 healthy volunteers (45 females; age between 18 and 31) were recruited for the study. The sample size for each experiment was decided *a priori* based on a series of power analyses (electronic supplementary material, Methods). All participants were right-handed, had normal or corrected-to-normal vision, and had no previous history of mental or neurological illness. The experimental protocol was approved by the Research Ethics Committee of University College London and adhered to the ethical standards of the Declaration of Helsinki. All participants were naive regarding the hypotheses underlying the experiment and provided their written informed consent before the beginning of the testing, after receiving written and verbal explanations of the purpose of the study. All participants received monetary compensation (£8 per hour) for their involvement in the study. The hypotheses, procedures and analyses of Experiment 3 were preregistered (https://osf.io/ygqnf).

### (b) Apparatus

Figure 1 shows a schematic representation of the experimental set-up. Participants sat at a desk, resting both their arms on the surface. A left cosmetic silicone glove (Realistic Prosthetics Ltd, model RPL 503/505, UK) filled with cotton wool was placed in front of the participants at approximately 20 cm [53] to the right of their left hand so to be aligned to their body midline. The participants' left arm and the robotic set-up were covered by a foamboard screen and remained unseen throughout the entire experiment. The silicone glove was instead clearly visible through an aperture in the foamboard. A desk lamp was used to illuminate the rubber hand under the foamboard.

The RHI was elicited using two identical brushes, following the classical RHI procedure [2,54]. The experimenter sat opposite to the participant and used the two brushes to stroke homologous points of the participants' left hand and the cosmetic glove (between the middle and the index finger) either synchronously (approx. 1 Hz) or asynchronously (approx. 1 Hz, 180° out of phase). The RHI stimulation lasted for 60 s [54]. To obtain an estimate of the participants' proprioceptive drift, a webcam was mounted on the ceiling above the set-up. The webcam provided a top view of the participants' arms and was used to take accurate measurements of the pointing movements made by the participants at the end of the RHI induction (figure 1). The coordinates of each proprioceptive judgement were extracted by each picture through the ImageJ software (http://rsbweb.nih.gov/ij/) and then converted from pixels to centimetres.

The sensorimotor self-touch stimulation was implemented using two six-degrees-of-freedom robotic arms (3D Systems, Geomagic Touch X, South Carolina, USA) linked in a computer-controlled leader-follower system (figure 1). In this system, any three-dimensional movement of the right-hand leader robot is reproduced by the follower robot with an estimated lag of approximately 2.5 ms [24,25]. A wooden rod with a rounded tip was attached to the follower robot in correspondence of the middle finger of the participants' left hand. Thus, the leader robot movements produced corresponding gentle strokes from the follower robot on the back of the participants' left middle finger. In order to keep the active self-touch condition as naturalistic as possible, we did not constrain the kinematics of participants' right-hand movements, or the number of strokes performed in each stimulation. To ensure that both motor and tactile signals were matched as closely as possible across the different conditions, prior to the beginning of the experiment, participants were instructed to perform their voluntary movements at the same pace as the passive movements produced by the experimenter. The participants then practised the movements under the supervision of the experimenter, who also checked the pace of participants' movements throughout the testing. This set-up allowed us to create a laboratory equivalent of ordinary tool-mediated self-touch. Importantly, the use of tool-mediated self-touch as opposed to skin-to-skin self-touch allowed us to make sure that the right-hand movement did not provide any spatial information about the position of the left hand.

### (c) Experimental design

Experiments 1 and 2 tested, respectively, whether self-touch has a restorative versus a protective effect on bodily self-awareness. We reasoned that if self-touch has a restorative effect on bodily self-awareness, a reduction of RHI effect should be observed when a brief self-touch stimulation is performed *after* the RHI induction (Experiment 1). Conversely, if self-touch has a protective effect on bodily self-awareness, a brief self-touch stimulation should reduce the participants' susceptibility to a subsequent RHI (Experiment 2). Both experiments had a 3 (visuo-tactile stimulation: synchronous, asynchronous, no stimulation) × 3 (self-touch condition: active, passive, no self-touch) within-participants design. The order of both factors was completely randomized across participants. Each of the nine possible combinations of these factors was repeated three times, giving a total of 27 trials per participant for a testing session of about approximately 90 min. Between trials, the participants were asked to take short breaks and to make large movements with their arms so as to cancel out any lingering effect from the previous trial.

Experiment 3 aimed to replicate the findings of Experiment 1 in a larger sample and to provide further experimental control conditions for the potential unimodal effect of right-hand movement and left-hand touch alone. The experiment had a 2 (visuo-tactile stimulation: synchronous, asynchronous) × 2 (movement: present, absent) × 2 (touch: present, absent) fully within-participants factorial design. Each of the eight combinations of these factors was repeated three times, for a total of 24 trials and a testing session of about approximately 90 min. Short breaks were introduced between trials to prevent any potential lingering effect from the previous stimulation.

### (d) Procedure

Each trial in each experiment consisted of a RHI induction phase and a self-touch stimulation phase which was performed either after (Experiments 1 and 3) or before (Experiment 2) the RHI (figure 1). Additionally, participants performed two pointing movements, one at the beginning of the trial (baseline pointing), and one at the end of the trial (late pointing). Before the beginning of each experiment, participants familiarized with the experimental set-up and received specific training for each phase of the experiment.

#### (i) Experiment 1

At the beginning of each trial, participants performed a baseline pointing movement. They were asked to close the eyes and to use their right index finger to produce a ballistic movement starting from the resting position on the desk and landing on the foamboard, in correspondence of the middle of their left wrist. Participants were asked to be as accurate as possible and to keep their finger on the foamboard for a few seconds to allow the experimenter to take a picture of their pointing through the webcam placed above the set-up. Next, in the RHI induction phase, participants were asked to open their eyes and to fixate the cosmetic glove through the aperture in the foamboard, while the experimenter performed either a synchronous or asynchronous RHI stimulation for one minute. In a third (control) condition, participants fixated the rubber hand for one minute without receiving any visuo-tactile stimulation. Right after, in the self-touch phase, participants were asked to grasp the handle of the leader robot with the right hand and then close their eyes. We designed our experiments so that the self-touch phase never contained any visual component, in order to ensure that only the sensorimotor signals arising from self-touch were available during this phase (figure 1). In the active self-touch condition, participants produced short (approx. 6 cm) back-and-forth movements on the proximo-distal axis. In the passive self-touch condition, participants grasped the robot handle with their right hand and then rested as passively as possible while the experimenter moved the handle of the leader robot in the same fashion as the active self-touch condition described above. In both conditions, the follower robot generated simultaneous and spatially corresponding tactile strokes on the back of participants' left middle finger. Either self-touch stimulation lasted for 15 s, as [54] showed a consistent presence of proprioceptive drift 15 s after a 60 s RHI. In a third (control) condition, no self-touch stimulation was provided, and the participants were asked to just wait for 15 s with their eyes closed before moving to the next phase of the trial. Finally, the participants were asked to close the eyes again and to perform another pointing movement to obtain an estimate of their proprioceptive drift.

To investigate participants' explicit judgements on the RHI, we also collected self-report measures of the participants' subjective experience in each experiment. At the end of the first trial of each condition, participants responded to several questionnaire items about the RHI [3]. The items of the questionnaire were adapted from [30] (electronic supplementary material). Participants rated their agreement with each item of the questionnaire on a scale from 0 (strongly disagree) to 6 (strongly agree). The ratings were then transformed to a scale from −3 to +3 for visualization purposes.

#### (ii) Experiment 2

Experiment 2 was identical to Experiment 1 in all respects, except that the order of the self-touch and the RHI phases was inverted, such that participants first performed one of the three self-touch stimulations and then experienced one of the three RHI conditions. As in Experiment 1, a baseline and a final pointing movement were acquired at the beginning and the end of each trial, providing an estimate of the participants' proprioceptive drift. RHI questionnaires were delivered at the end of the first trial of each condition.

#### (iii) Experiment 3

In Experiment 3, the order of the self-touch and RHI phases was the same as in Experiment 1. However, the active and passive self-touch conditions were replaced by a factorial combination of movement (present, absent) and touch (present, absent). The conditions where movement and touch were both present or both absent were identical, respectively, to the active self-touch and no self-touch conditions in Experiment 1. The other two factorial combinations (movement only and touch only) served as control conditions to investigate whether either component of self-touch was sufficient to mediate any effect on bodily self-awareness. In the movement only condition, participants held the handle of the leader robot with their right hand and performed the same proximo-distal movements described above. Crucially, the follower robot was disconnected from the leader-follower system in this condition, such that the participants' movement did not produce any tactile stimulation. In the touch only condition, instead, the participants were asked to close their eyes and rest their hand on the desk while the experimenter moved the follower robot. This produced a tactile stimulation on the participants' left hand in absence of any right-hand movement. As in the other experiments, a baseline and a final pointing movement were acquired at the beginning and the end of each trial, providing an estimate of the participants' proprioceptive drift. RHI questionnaires were delivered at the end of the first trial of each condition.

### (e) Statistical analysis

We operationalized participants' bodily self-awareness in terms of proprioceptive drift [2,4], defined as the perceived position of the participant's real left hand on the mediolateral axis. This measure was acquired as a series of independent proprioceptive judgements, performed through pointing movements. The proprioceptive drift was expressed as the difference between the perceived position before the start of the trial (i.e. baseline) and the one expressed after each RHI and self-touch manipulation. Thus, a positive proprioceptive drift corresponds to a shift in perceived position of the hand to the right, i.e. towards the location where the rubber hand was seen.

To test our hypothesis that active self-touch has a restorative (Experiment 1) or protective (Experiment 2) effect on bodily self-awareness, we ran two separate 3 (visuo-tactile stimulation: synchronous, asynchronous, no stimulation) × 3 (self-touch condition: active, passive, no self-touch) repeated measures ANOVAs on the proprioceptive drift scores and each question of the self-reports of all participants in Experiments 1 and 2. In Experiment 3, we ran a 2 (visuo-tactile stimulation: synchronous, asynchronous) × 2 (movement: present, absent) × 2 (touch: present, absent) repeated measures ANOVA on the proprioceptive drift scores and each question of the self-reports. When the data violated the sphericity assumption, a Greenhouse–Geisser correction was applied. ANOVAs were followed up by Bonferroni corrected *post hoc* pairwise comparisons.

## Data Availability

All data needed to evaluate the conclusions in the paper are present in the electronic supplementary material and in the accompanying Open Science Framework repository (https://osf.io/ygqnf). Supplementary material is available online [55].

## Classification

Biological Sciences/Neuroscience.
